# Design and Development of an AIoT Architecture for Introducing a Vessel ETA Cognitive Service in a Legacy Port Management Solution

**DOI:** 10.3390/s21238133

**Published:** 2021-12-05

**Authors:** Clara I. Valero, Enrique Ivancos Pla, Rafael Vaño, Eduardo Garro, Fernando Boronat, Carlos E. Palau

**Affiliations:** 1 Communications Department, Universitat Politècnica de València, 46022 Valencia, Spain; ravagar2@upv.es (R.V.); fboronat@dcom.upv.es (F.B.); cpalau@dcom.upv.es (C.E.P.); 2 Prodevelop S.L., 46003 Valencia, Spain; eivancos@prodevelop.es (E.I.P.); egarro@prodevelop.es (E.G.)

**Keywords:** maritime logistics, automatic identification system, artificial intelligence, internet of things, artificial intelligence of things

## Abstract

Current Internet of Things (IoT) stacks are frequently focused on handling an increasing volume of data that require a sophisticated interpretation through analytics to improve decision making and thus generate business value. In this paper, a cognitive IoT architecture based on FIWARE IoT principles is presented. The architecture incorporates a new cognitive component that enables the incorporation of intelligent services to the FIWARE framework, allowing to modernize IoT infrastructures with Artificial Intelligence (AI) technologies. This allows to extend the effective life of the legacy system, using existing assets and reducing costs. Using the architecture, a cognitive service capable of predicting with high accuracy the vessel port arrival is developed and integrated in a legacy sea traffic management solution. The cognitive service uses automatic identification system (AIS) and maritime oceanographic data to predict time of arrival of ships. The validation has been carried out using the port of Valencia. The results indicate that the incorporation of AI into the legacy system allows to predict the arrival time with higher accuracy, thus improving the efficiency of port operations. Moreover, the architecture is generic, allowing an easy integration of the cognitive services in other domains.

## 1. Introduction

Maritime transport is one of the main pillars of world trade and economy [1,2]. It is a complex environment where logistic planning has a fundamental role to ensure an efficient use of resources [3]. The maritime community is composed of a large number of actors responsible for different activities related to port management; port authorities need open and neutral platforms that connect multiple systems, and these are known as port community systems [4]. They allow safe and intelligent exchange of information between different organizations that comprise the whole maritime community. This exchange enables the optimization, management, and automation of logistics and port processes [5], thereby improving the efficiency and thus the competitiveness of port communities. These shared and organized platforms are used both by public and private stakeholders [6] such as shipping agents, customs, freight forwarders, and terminal operators.

Seaport terminals can be considered one of the most important parts of the logistics network, because they are located in the center of the supply chain [6,7] connecting the land-side and seaside, as well as the export and import sides of the supply chain. Therefore, it is of great importance to guarantee a fluid operation of the terminal to ensure the efficiency of the entire supply chain [8]. To this end, the calculation of an accurate estimated time of arrival (ETA) of ships at ports is a key aspect to optimize time, resources, and costs of maritime operations [9]. The operations to be carried out by the different actors in the chain when a vessel arrives at a port are planned based on this date/time, so any deviation between the estimation and the reality can generate delays, thus affecting the rest of the chain. Such deviations may result in contracted services along the logistics chain not being available as planned, causing delays in the logistics chain and consequently increasing financial costs. Examples of this impact are the scheduled quay not being available because the terminal is operating another vessel or the vessel not being able to dock due to coincidence with the maneuvers of another vessel.

Another important system of this logistic chain is the automatic identification system (AIS) [10]. It is a mandatory international navigation safety communications system, under the provisions of the Safety Of Life At Sea (SOLAS) conventions endorsed by the International Maritime Organization (IMO). AIS emerged with the intention of preventing collisions at sea, complementing other existing solutions such as radar and other regulated means in the Convention on the International Regulations for Preventing Collisions at Sea (COLREG). This system allows a vessel to be identified by a unique identifier, MMSI (maritime mobile service identity), and is mandatory for all vessels exceeding 300 gross tonnage. AIS provides information about vessels, such as the vessel name, MMSI, current position and destination, ETA, course, or speed.

The ETA is calculated by a maritime operator using limited sources of information and a very basic estimation technique based on simple arithmetics [11], and it is unable to provide high accuracy [12] or to include other relevant factors beyond the start travel time and distance to port. The estimated ETA is manually inputted into the AIS communication system from where the port obtains the ETA. These data generally differ considerably from the actual time of arrival (ATA), becoming thus a rough estimation [13]. Examples of these setbacks are changes in vessel speed or adverse weather conditions, which are not considered in the calculation. A deviation between the ETA and the ATA implies high economic cost, as it leads to underutilization of resources or not having the necessary ones at the required time. These issues could be avoided with a more accurate arrival prediction.

The last few years have seen accelerated growth in the field of artificial intelligence [14,15]. More specifically, the application of AI techniques for maritime process improvement has grown considerably. Several studies [16,17] show that it is possible to use AIS data as historical maritime traffic data patterns. These patterns enable us to convert the raw data into decision-supporting information to allow maritime anomaly detection and vessel route prediction. Moreover, estimating the vessel arrival time in port areas by exploiting historical vessel tracking data is a popular topic that has motivated a lot of research [18,19,20,21,22,23]. However, very few port solutions currently incorporate these AI techniques. Most of them are deterministic in nature, providing recommendations based on predetermined rules that take action based upon the occurrence of a specific event. The validation of these models in real systems remains an underexplored field.

On the other hand, in order to automate and provide better solutions for decision making, the integration of machine learning is becoming essential for companies. Consequently, the platforms and frameworks that provide this type of service have been evolving, providing more sophisticated ML services. The most relevant are IBM Watson [24], Google AI [25], Microsoft Azure [26,27], and Amazon AWS AI [28]. These platforms provide design automation tools and predefined modules, as well as testing and implementation of automatic learning services, which makes them ideal environments for companies that do not have sufficient resources or the necessary experience. Nevertheless, they present limitations regarding interoperability, extensibility, and cost. Moreover, it is important to highlight that there are not open source platforms that incorporates jointly IoT and AI.

This work has been designed around the following research questions:

- RQ1. How to extend an IoT architecture to integrate in it cognitive services?
- RQ2. Is it possible to create and integrate in a commercial legacy system a reliable cognitive service based on ML algorithms that gives an accurate prediction of a vessel’s ETA using IoT data sources?
- RQ3. Which IoT data sources provide the greatest feature importance?

To answer these questions, this paper proposes an artificial intelligence Internet of Things (AIoT)-based open-source architecture that will enable the incorporation of cognitive services into legacy IoT systems that lacks of these capabilities. The manuscript not only proposes the AIoT-based architecture but also subsequently implements it and validates it over a FIWARE-based legacy sea traffic management IoT platform. The vertical specific solution is capable of producing reliable ETA predictions for vessel transport. For this purpose, several ETA algorithms and models proposed in the research literature have been analysed in order to identify which provides the best results in this specific scenario, and allows us to further improve the decision support offered by the legacy solution.

The starting point is a legacy real-time vessel activity monitoring system that allows a port to manage and optimize maritime activities related to the flow of vessels. The port service includes a graphical user interface (GUI) that displays information of vessels located within range of an AIS antenna located in the vicinity of the port. Following the current tendency of combining AI and IoT to enable IoT cognitive networks and systems [29], it is intended to demonstrate that it is possible to integrate a predictive model within the commercial system and thus further improve with machine learning (ML) the current estimations of the port service offering a better decision support. Using the data of the system, gathered by means of Internet of Things (IoT) technology, the cognitive service should be able to interpret and evaluate the status of the whole IoT system in near real time and in an automatic way, thus making accurate ETA predictions that allow managing port resources more efficiently. These data sources will be handled by a European cloud IoT platform created by the European Commission called FIWARE. The cognitive service will be incorporated in the legacy system using the proposed AIoT architecture. To support advance AIoT data interpretation, the architecture will incorporate a FIWARE cognitive component. The introduction of this cognitive service will allow to extend the lifetime of the legacy solution.

The remainder of the paper is structured as follows. The proposed AIoT system architecture is presented in Section 2. Section 3 includes a discussion of the results obtained. Finally, concluding remarks are given in Section 4.

### Research Contributions

This work provides a first open source AIoT approach that enables the extension of IoT-based applications to incorporate AI, ensuring compliance with FIWARE guidelines and standards. The modular approach together with standardization and open source will allow developers to add new cognitive services to the platform according to their needs.

The paper describes the design and implementation process of the proposed architecture in a commercial marine legacy system. Marine professionals can use the steps followed in this paper to incorporate a cognitive service with similar characteristics into their legacy IoT systems, avoiding the migration of commercial AIoT systems available in the market (e.g., IBM Watson, Google AI, or AWS AI). Other professionals can benefit from a novel generic approach to embedding cognitive services in an open-source IoT platform and use this approach in any type of industrial domain.

The objective of this work is not to advance the state of the art of the vessel ETA models, but rather to apply and validate them in a real port management solution, jointly using AI and IoT. According to previous work, it is considered that AIS and maritime oceanographic data are sufficient for the system to provide reliable forecasts. Thus, the predictive algorithm contained in the cognitive service will be trained using both data sources, AIS data and oceanographic data. On the one hand, this will permit us to analyse whether these data sources are sufficient to build with it a reliable prediction system in a port environment, and on the other hand, whether the two data sources have the same weight in the prediction of the vessel ETA in this concrete scenario.

The major contribution of the proposed architecture is the design, development, integration, and validation of a cognitive component responsible for the inclusion of cognitive services in the FIWARE ecosystem. Thanks to the combination of AI and IoT, the cognitive service is expected to be able to interpret and evaluate the state of the entire system in near real time and automatically. To the best of the author’s knowledge, this study presents the first component of the FIWARE IoT platform that allows the creation of solutions based on AI for the ecosystem, while ensuring that FIWARE guidelines and standards are met.

## 2. Materials and Methods

### 2.1. Methodology

This work has been carried out in collaboration with a private company and therefore seeks to achieve an operational result. The methodology followed uses the Volere requirements specifications [30] while applying the DSR method [31] following the Hevner guidelines [32]. The methodology used to define, implement, and validate the system comprises the following phases: problem identification and work objectives establishment, requirements elicitation, system architecture design, development process, system component integration, and system validation.

#### 2.1.1. Problem Identification and Work Objectives Establishment

The research questions presented above are key to identifying the problems to be solved and establishing the objectives of this study. Before the COSIBAS project started the problems and limitations of the current port management solutions were identified and the main objectives of the work were established to respond to the identified research questions (Section 1).

#### 2.1.2. Requirements Elicitation

First, functional and non-functional requirements were identified. To this end, the different needs of the actors who interact with the system—the administrators and the end users—were taken into account. Volere [30] templates have been used to define the requirements. Functional requirements were collected from the analysis of the different use cases of the system. Among the requirements defined, one of the most remarkable is the scalability of the architecture. It should allow the introduction of as many cognitive services as desired without making significant changes to the system.

#### 2.1.3. System Architecture Design

In terms of design, a study of the state of the art and limitations of the different IoT architecture, frameworks, as well as AI solutions was carried out. After a deep analysis, FIWARE was selected as the IoT platform of the system. This choice was motivated by the multiple benefits of the platform such as high scalability, interoperability, and robustness, since its architecture is based on micro-services. Moreover, FIWARE is an open source platform with a strong and active community.

After the selection of the platform, components, guidelines, and FIWARE, standards were examined in order to design a compliant system architecture (Section 2.2). The conceptualization of the AIoT architecture included the design of the general workflow (Section 2.2.2) and data model of the messages to be exchanged between the system components. This design included the internal messages to be exchanged in the cognitive component, defining how it communicates with the rest of the system’s components following a decoupled approach.

#### 2.1.4. Development

After the design of the architecture, its components were developed. The cognitive component was developed ensuring that FIWARE NGSI standards for interoperability were met. It was developed in Python 3 and subsequently containerized using Docker [33], the packaging method used by FIWARE, ensuring a single deployment of the entire solution.

The development of the predictive model was the last step of development, as initially, no training dataset was available. In order to train the algorithm used for the cognitive service, two datasets were created. The first dataset consists of 700,614 records of AIS data from an AIS antenna located close to the port of Valencia. It includes information from the routes followed by 271 vessels to reach the port in a time range between 11 May and 24 June 2021. The second dataset includes marine weather information corresponding to the geolocation of the vessels during this period. The dataset has been constructed using the provider World Weather Online [34] and includes 1548 records.

During the cognitive service exploratory data analysis, all records that contain missing or incorrect data have been removed. Furthermore, the value of some of the AIS features also had to be standardised. Since AIS data do not provide the vessel’s ATA, the value needed to train the algorithm included in the cognitive service, it has been calculated by storing the timestamp of the vessels when they cross a certain area of the port of Valencia.

The most relevant features have been identified to be used for model building. It is important to note that irrelevant and redundant features can confuse a learning algorithm by making it difficult to distribute the small set of truly relevant features [35]. Therefore, it is of great importance to identify which features provide the most information for prediction and only include these in the model. The data have been divided into training and validation sets. This is not done randomly, but each dataset includes complete vessel routes. In this way, the algorithm is trained using a subset of the routes and is validated using different ones. This ensures correct validation of the model. Finally, the ETA prediction model has been created by training several regression algorithms (such as decision tree, support vector regression, random forest, or K-nearest neighbors) with the objective of predicting the minutes remaining for the vessel to arrive at its destination.

#### 2.1.5. Integration

During the integration, data sources needed to feed the cognitive service were introduced in the system. In parallel, the developed prediction model was serialized using the Python pickle library [36] to allow its integration with the cognitive service. Finally, the communications of the components of the architecture were established using their REST APIs.

#### 2.1.6. Validation

The system has been validated considering the environment of the port of Valencia, where the commercial sea traffic management software Posidonia Operations [37] has been integrated with the proposed architecture. Posidonia Operations is an integrated port operations management system that enables a port to optimise maritime activities related to the flow of vessels within the port’s service area, integrating all stakeholders and all relevant information systems. Posidonia has its own GUI, so the cognitive service has been validated using this solution together with its interface.

The validation of the architecture components was performed as the components were developed. The validation of the integration between the different components of the system was carried out through integration tests using the API REST of the components. Once all the communications between the different components were established, the interface of the legacy solution was modified to link it with the cognitive service. Once this was achieved, the system was validated together with the interface.

The validation of the developed cognitive service has been performed using the mean absolute error (MAE). MAE illustrates the average magnitude of error between the predicted and measured vessel time of arrival. The definition of MAE and is given by:

(1) $$\mathrm{MAE}=\frac{\sum_{i=1}^{n}\left|y_{i}-x_{i}\right|}{n},$$

where $x_{i}$ are the actual values and $y_{i}$ the predicted ones from the n records considered. The results of the validation are included in Section 3.

### 2.2. System Architecture

**Figure 1:**
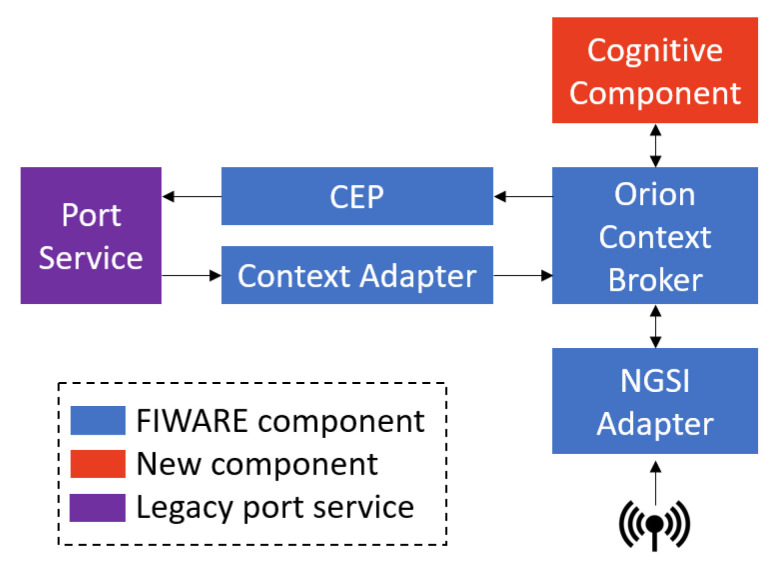
Simplified system architecture.

Existing FIWARE components do not allow to create and integrate a vessel ETA cognitive service in the port service. A new element, the cognitive component, has been defined and incorporated for this purpose, ensuring that FIWARE standards are complied with. The six functional blocks of the proposed system architecture (Figure 1) are briefly described below:

- The port service is a real-time vessel activity monitoring system that detects multiple events in the life cycle of a vessel in port and allows us to automate actions and assist a port operator in controlling the vessel’s visit to the port.
- AIS and Weather NGSI adapters have been developed to insert in the system the AIS data gathered by the antenna and the weather conditions.
- Orion Context Broker (OCB) works as an aggregator of context data and, at the same time, is an interface between the components of the architecture. Hence, the other elements of a FIWARE system can publish or consume data without having specific knowledge about the rest of the system.
- A specific context adapter has been developed for this scenario. It is responsible for ensuring that data coming from the port service are transformed to be compliant with the FIWARE NGSI standard. It handles redirected requests (updateContext) and notification requests (notify) sent by the OCB, transforming them into requests to the web interface.
- The cognitive component offers services based on the use of cognitive algorithms. This element retrieves data from a variety of sources and is able to send the results and decisions to other FIWARE components. ML algorithm-based models are embedded in this component to give rise to an AIoT system.
- A complex event processor (CEP) analyzes event data in real-time, detecting patterns in the incoming events. The CEP can receive events from different event producers of the FIWARE platform. In this case, the CEP analyzes the difference between the planned ETA and the calculated ETA. Depending on the degree of deviation between both values, it will send a notification, warning, or alarm to the port service.

For the implementation of the proposed architecture, FIWARE guidelines and standards have been followed. The introduction of a new component is complying with the component-based nature of FIWARE pillars. Moreover, its integration is simplified, since all components comply to the FIWARE NGSI standard interface.

#### 2.2.1. Cognitive Component

The cognitive component (mark in red in Figure 1) has been designed to support the evolution from large-scale heterogeneous IoT-based systems to AIoT systems. The cognitive component communicates with OCB, the core of the FIWARE platform, using the standard FIWARE NGSI API that provides the basis for interoperability and portability with FIWARE. The cognitive component consists of a central element, the cognitive enabler, and as many cognitive services as desired.

The cognitive enabler is a transversal and common component responsible for managing communications between different cognitive services and OCB. The cognitive enabler receives cognitive requests coming from OCB and forwards them to the appropriate cognitive service. In the reverse direction, it receives response messages from cognitive services that it routes to OCB. Thus, the cognitive enabler decouples the communications performed between OCB and the cognitive services, allowing an easier scaling of the cognitive services.

Cognitive services are a set of microservices that include previously trained ML algorithms. Using NGSI data model, they return a prediction to the cognitive enabler, which is responsible for entering it into OCB. The cognitive request from the cognitive enabler includes the OCB id entity, or entities that contain the input features needed to make the predictions. The acquisition of these features, stored in OCB, is handled by the cognitive enabler. When the features are obtained and entered into the model, a prediction is obtained, which is stored in OCB via the cognitive enabler.

It is important to note that the presented architecture is generic and scalable. It allows the incorporation of as many cognitive services as desired, leaving the cognitive enabler to handle the message flow.

#### 2.2.2. Message Flow

**Figure 2:**
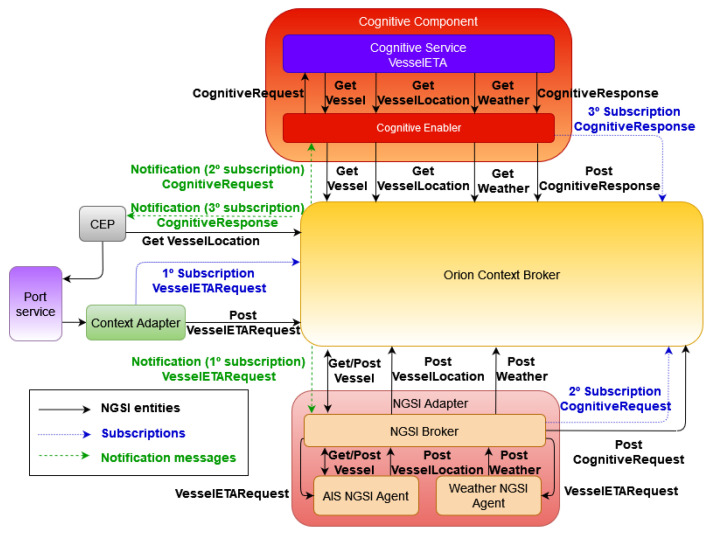
System architecture and messages.

The presented architecture (Figure 2) manages communication between its components using the FIWARE asynchronous notification mechanism. Its main benefit is that it allows us to alleviate network traffic, improving system performance and allowing the cognitive component to be notified by OCB of changes made in the context. Similarly, this functionality allows the cognitive component to notify OCB of changes made to the context information, and this, in turn, to other components, such as the CEP. Therefore, the cognitive component can act as a publisher/consumer of the context information.

**Figure 3:**
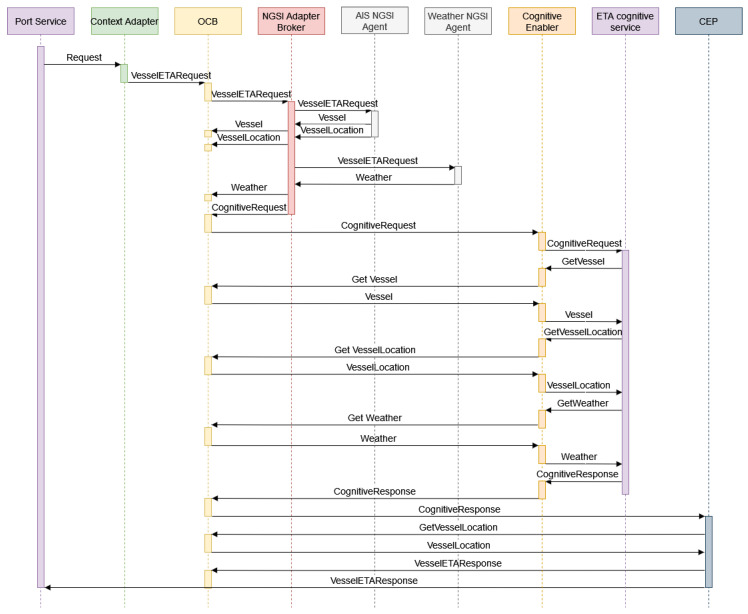
Message flow.

Figure 3 summarizes the architecture message flow. When a user requests to calculate the ETA of a vessel in the graphic application (port service), the context adapter transforms the user’s request into an NGSI entity of type VesselETARequest. Before sending this new entity to OCB, it is checked if there is a subscription associated with this type of entity. If not, a subscription that notifies the context adapter of this new entity is created.

When the NGSI broker receives a VesselETARequest notification, is forwarded to the NGSI agents. The AIS NGSI agent uses the maritime mobile service identity (MMSI) field to query AISHub API to obtain the vessel information. Static vessel information (MMSI, name, flag, vessel type, among others) is included in a NGSI entity of type vessel, while dynamic information of the vessel (location, course, speed, ETA, and so on) is included in a VesselLocation entity. If there was already information about this vessel (identified by its MMSI) in OCB, its associated entities are updated instead of creating new ones. In pararell, the weather NGSI agent obtains marine weather information corresponding to the vessel’s geolocation, creating a weather entity. The NGSI agents are responsible for creating the NGSI entities, and the broker is in charge of entering them in OCB. Subsequently, the NGSI broker creates an additional entity of type CognitiveRequest (see Appendix A). It includes the identifiers of the Vessel, VesselLocation, and weather entities, in addition to a field that indicates the location of the specific cognitive service to be used. Before inserting the CognitiveRequest entity in OCB, it is checked whether a subscription for this type of entity exists in OCB. If it does not exist, a subscription is created to notify the cognitive enabler every time an entity of type CognitiveRequest is created.

When the cognitive enabler receives a notification, the entity contained in the notification is examined and extracted. Then, the identifiers of the vessel, VesselLocation, and weather type entities are forwarded to the vessel ETA cognitive service, which receives the request through its API. Since the information associated with the received entities is stored in OCB, the service makes a request to it through the cognitive enabler to obtain the entities information. After that, the ETA cognitive service extracts from the entities the necessary features that the model needs and performs the prediction. The last remaining step in the service consists of sending the predicted ETA back to the cognitive enabler. The service inserts the predicted ETA into a new NGSI entity, the CognitiveResponse (see Appendix A), sending it to the cognitive enabler. Before inserting the entity in OCB, the cognitive enabler checks that there is a subscription of type CognitiveResponse that notifies the CEP. If it does not exist, it creates this subscription.

When the CEP receives a notification, it makes a request to OCB to obtain the VesselLocation entity of the associated vessel where the planned ETA is included. Then, it compares the predicted ETA (value included in the received notification) with the planned ETA. Depending on the difference, a response is created, the type of which can be informative or warning with a description. This response is packaged in an entity of type VesselETAResponse, which is, on the one hand, sent to OCB for insertion in OCB, and on the other hand, sent to the port service.

## 3. Results and Discussion

The framework has been tested by integrating a vessel ETA cognitive service in the Posidonia operations [37] legacy system using the port of Valencia. According to PortEconomics [38], this is the main Spanish port and the fifth in Europe regarding transport of containers, managing a volume of around 5.4 twenty-foot equivalent unit (TEUs) in 2020.

**Figure 4:**
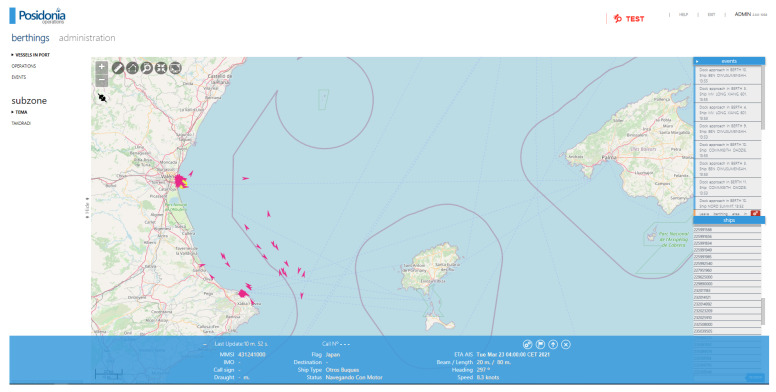
Port service user interface.

Figure 4 shows the port service GUI. The icons corresponding to the vessels are displayed using the information provided by an AIS Dispatcher, an AIS data-forwarding utility that receives AIS data via UDP stream or TCP connection. By means of the geographical information system software integrated in Posidonia operations [37], this component reads the information received by the AIS antenna and positions the vessels in a map. The location and direction of the ships are represented by an arrow. Furthermore, if the information is available in the AIS data, the interface displays the following information for the selected vessel: MMSI, IMO, call sign, draught, flag, destination, ship type, status, planned ETA, beam, length, heading, and speed.

**Figure 5:**
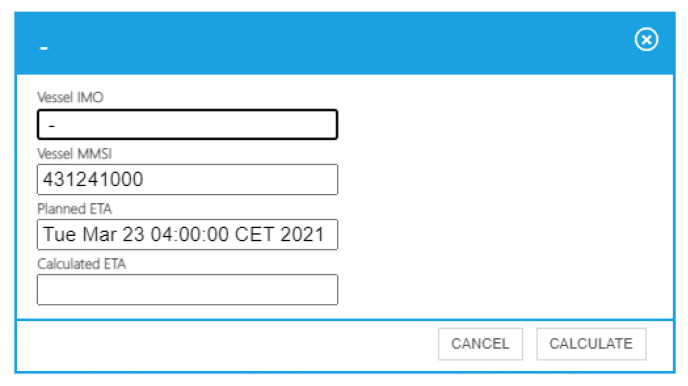
Basic vessel information.

**Figure 6:**
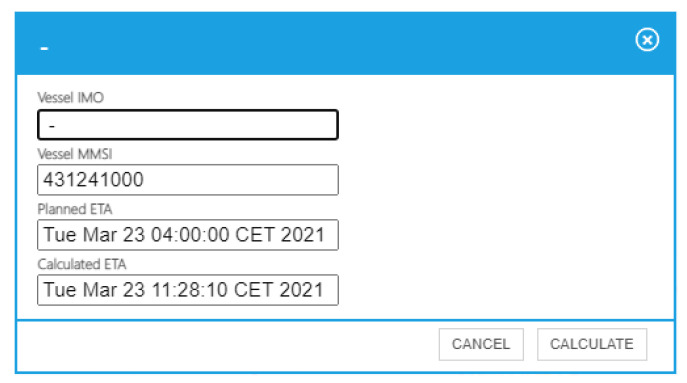
Predicted ETA.

When a user selects a vessel, a pop-up window appears (Figure 5). This new window has been developed to allow the user to request and show the ETA prediction of an specific vessel. By clicking on the calculate button, the user initializes the message flow described in Section 2.2. The context adapter is responsible for transforming the user’s request into an NGSI entity that initiates the flow that triggers the calculation of the ship’s arrival time at its destination by means of the vessel ETA cognitive service. When the message flow ends, the interface receives a notification from the CEP and displays the prediction (Figure 6).

In relation to the development of the predictive model of the vessel ETA cognitive service, the used features and their importance are presented in Table 1. The feature importance analysis revealed that the use of marine weather conditions information did not improve the predictive performance of the model. The analysis positions the wind speed as the most significant of all the oceanographic features, but still, its model contribution is minor compared to the selected features. This is because the weather conditions are already reflected in the speed over ground (SOG) feature. It is the captain of the vessel who determines the navigation according to the weather conditions. If the weather is unfavourable, the ship reduces its speed. So, the oceanographic variables only add redundancy and unnecessary complexity to the model, which makes its behaviour worse. Consequently, as oceanographic data is not needed, the costs associated with using the provider World Weather Online [34] were discarded.

**Figure 7:**
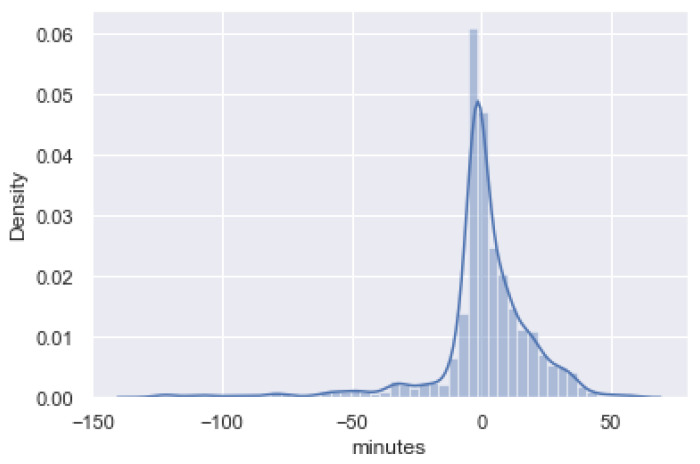
Error distribution.

MAE formula (presented in Section 2.1.6) has been used to validate the new functionality added to the legacy system. The starting point was a scenario were the MAE of the ETA provided by AIS data (without using the cognitive service) was 1066 min. After the development and integration of the cognitive service into the legacy system, a high ETA estimation was achieved. Of all the regression algorithms used during training, the KNN regression algorithm presented the best results, with a MAE of 13 min. However, although the MAE allows us to evaluate the performance of the model, more detail can be obtained by plotting the error between the predicted value and the actual value in a scatter plot (Figure 7). The plot shows the desired behaviour. The bell is narrow and centred on zero. This indicates that most of the errors that occur are close to zero. The worst, very residual predictions estimated the ship’s arrival time with an error of 50 min.

If more training data were available, it would be possible to further improve. This is outside the scope of this study, whose main objective is to present an AIoT architecture that allows the incorporation of a cognitive service in a legacy port management solution to manage the port terminal’s resources more efficiently. The predictive model developed and integrated into the architecture through the cognitive enabler significantly improves the prediction of ship arrivals at the port. Furthermore, the followed component approach facilitates the integration of the developed predictive model in other systems.

## 4. Conclusions

This manuscript proposes a reference open artificial intelligence Internet of Things (AIoT) architecture, which has been designed by being compliant with the state-of-the-art FIWARE IoT framework specifications. To the extent of the author’s knowledge, although there are several AIoT commercial systems available in the market (e.g., IBM Watson, Google AI, or AWS AI), this work represents the first open-source AIoT approach that allows the creation of solutions based on AI, by ensuring that FIWARE guidelines and standards are met. To do so, a new component, namely cognitive component, has been introduced in comparison with a basic IoT FIWARE system. This component would be responsible of facilitating the incorporation of the cognitive services in the architecture following FIWARE’s philosophy. By making use of FIWARE’s asynchronous notification mechanism, any practitioner would be able quickly integrate the proposed cognitive component into its legacy IoT system.

Moreover, answering RQ1, the proposed AIoT architecture has been prototyped and validated for a real maritime legacy IoT system. In particular, cognitive capabilities with regards to the improvement of vessels’ estimated time of arrival (ETA) to any port stakeholder involved in the logistics supply chain has been incorporated. The results of the validation of the cognitive service are satisfactory, as they show a significant improvement in the estimation of vessel arrival times (answering RQ2), as well as confirm previous research analysis conclusions with respect to the irrelevance of oceanographic data for a proper ETA estimation. The latter provides an answer to RQ3. The cognitive ETA capabilities will lead to better planning of port activities, providing a reliable decision support system and thus managing the resources more efficiently. Marine sector practitioners can find in this work the procedures to be followed to incorporate a vessel ETA cognitive service using ETA prediction algorithms, while other professionals can benefit from a novel generic approach to embedding cognitive services in an open-source IoT platform.

The proposed architecture has been successfully validated in a commercial solution deployed in the port of Valencia. After this first experiment, it is planned to integrate a vessel ETA cognitive service in another Spanish port legacy system and possibly in a European port following the approach described. This work does not provide improvements with respect to the training of the ETA prediction algorithms. Following the current state of the art, these will have to be retrained specifically for the other ports. Nevertheless, the cognitive component will facilitate the incorporation of cognitive services, saving time in development and integration with legacy systems.

Further validation of the proposed architecture in other verticals is needed. It is important to stress that, although during this work only one cognitive service has been developed and integrated in a port management solution, the architecture is designed to incorporate cognitive services from any domain (health, transport, smart cities, etc.) within the FIWARE ecosystem. However, although the proposed architecture has been designed to be compatible with any domain, in this specific work, it has not been validated in other verticals. After that, the proposed FIWARE-based AIoT architecture in conjunction with the developed cognitive component will make it possible to extend the effective lifetime not only of legacy port IoT solutions without incurring in significant increase of operational costs, but also to any industrial domain (e.g., health, transport, smart cities, etc.) within the FIWARE ecosystem.

## Figures and Tables

**Table 1:** Features used in the best performing model and their importance.

Feature	Importance
Distance	0.188072
Longitude	0.183474
Latitude	0.167127
SOG	0.142248
COG	0.128106
Heading	0.103996
Draught	0.086977

**Figure A1:**
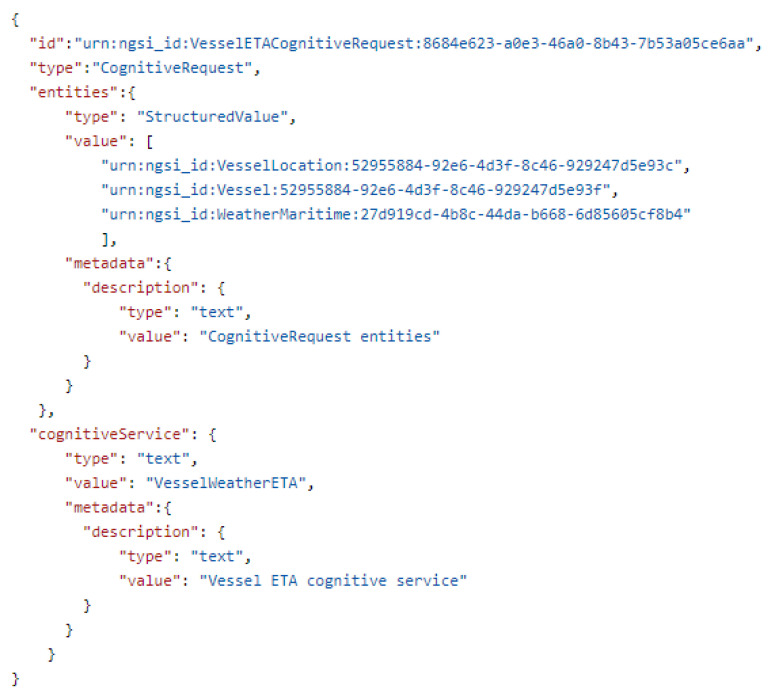
Cognitive Request message.

**Figure A2:**
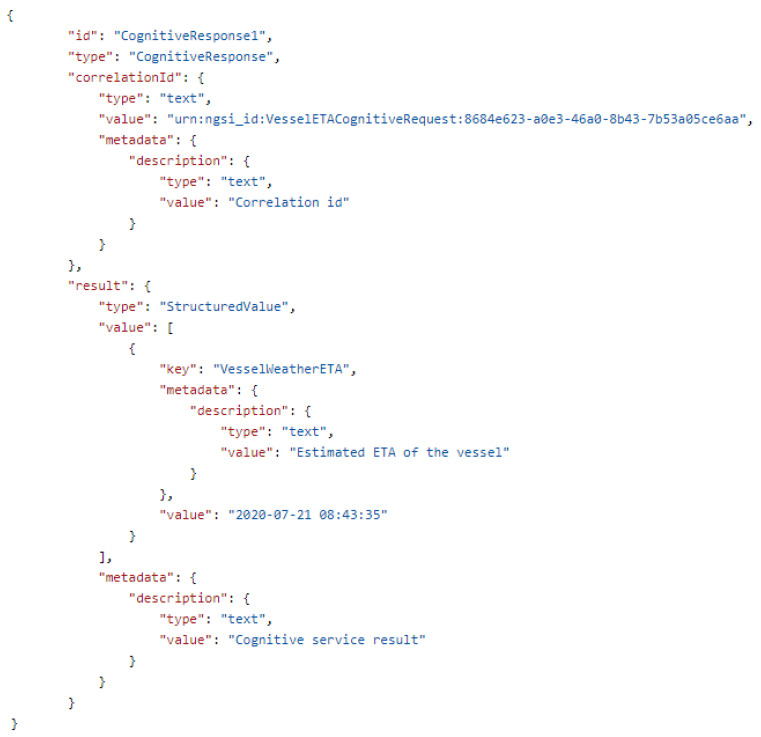
Cognitive Response message.

## Appendix A

This appendix refers to the data models defined and used in this work in the architecture design.

(i) Example of CognitiveRequest JSON message.

(ii) Example of CognitiveResponse JSON message.
